# Mapping Infodemic Responses: A Geospatial Analysis of COVID-19 Discourse on Twitter in Italy

**DOI:** 10.3390/ijerph22050668

**Published:** 2025-04-24

**Authors:** Gabriela Fernandez, Siddharth Suresh-Babu, Domenico Vito

**Affiliations:** Metabolism of Cities Living Laboratory, Center for Human Dynamics in the Mobile Age, Department of Geography, San Diego State University, 5500 Campanile Drive, San Diego, CA 92182, USA; siddharthsb06@gmail.com (S.S.-B.); dvito@sdsu.edu (D.V.)

**Keywords:** infodemiology, COVID-19 misinformation, geospatial analysis, public perception, social media analysis, Italy, Twitter

## Abstract

The COVID-19 pandemic intensified concerns about misinformation, sparking interest in the field of infodemiology, which examines the spread and impact of information on public health perceptions. This research examines how geographic location influenced COVID-19 discourse across 10 Italian cities by analyzing geographically tagged Twitter data. Our network analysis of 4792 high-degree nodes identifies key information spreaders and community structures, while spatiotemporal mapping reveals regional variations in information patterns and influential narratives. Results demonstrate significant geographic and cultural influences on public discourse. In Milan and Rome, economic and political narratives dominated, suggesting targeted messaging about economic recovery and government transparency. Southern regions like Naples require trust-building through community-led initiatives addressing cultural health beliefs. The study identified a clear dichotomy among influencers: established public figures provided evidence-based information, while another group cultivated followings through conspiracy theories, creating echo chambers for skeptical views. This research informs strategies for location-specific information campaigns, helping public health agencies combat misinformation more effectively. Findings emphasize the need for context-specific interventions that consider geographic, cultural, and socioeconomic factors to enhance community resilience during health emergencies.

## 1. Introduction

The COVID-19 pandemic underscored the pervasive challenge of misinformation in public health crises, drawing attention to the emerging field of infodemiology. This field explores how information both accurate and inaccurate affects public understanding, behavior, and response to health threats. During the pandemic, social media platforms like Twitter played a dual role, acting as conduits for real-time health updates while also serving as channels for misinformation. Research has shown that misinformation can distort public perception, fuel vaccine hesitancy, and hinder effective crisis response [1,2]. In contrast, these platforms have also been used to counteract misinformation, foster community support, and disseminate accurate information [3]. While many studies have examined misinformation globally, regional variations remain underexplored, especially in terms of how local contexts shape public perception. In Italy, where COVID-19 severely impacted different regions at varying times, understanding public responses to information based on geographic context is critical. Recent studies suggest that regional search behaviors and public discourse reflect distinct “infodemic” attitudes, indicating that cultural, media, and socioeconomic factors may influence the spread and impact of misinformation [4].

This study aims to analyze geographically tagged Twitter data from 10 Italian cities in the north, center, south, and islands to examine regional variations in public perception and information sharing and reception between northern urban centers like Milan, central regions including Rome, and southern communities such as Naples. We examine the geographic dimensions of COVID-19 discourse across Italian cities. Using a mixed methods approach that includes network analysis and spatial mapping, we seek to understand the prevalent narratives and how local contexts shape responses to COVID-19 information. Our findings are expected to illuminate how misinformation is perceived across diverse regions, informing more targeted public health communication strategies, leverage trusted regional influencers, and consider cultural context to mitigate misinformation and enhance resilience in future health crises.

## 2. Literature Review

The COVID-19 pandemic has intensified global concerns regarding the spread of misinformation, especially on social media platforms where information, both accurate and misleading, circulates rapidly. The study of information dissemination during health crises has led to the growth of “infodemiology”, a field that investigates the flow and impact of information on public health [5]. Infodemiology research became especially relevant during COVID-19, as misinformation related to the virus, vaccines, and public health measures surged, often influencing public attitudes and behaviors. Social media platforms, particularly Twitter, emerged as significant vectors for both the spread of misinformation and efforts to counteract it [6]. Twitter’s role as a public forum has allowed researchers to study the ways in which users discuss, share, and react to information about COVID-19, making it an invaluable source for infodemiological analysis.

Previous studies have highlighted how misinformation can undermine public health efforts, fueling vaccine hesitancy, conspiracy theories, and distrust in health authorities [7]. These findings have spurred initiatives to combat misinformation by using fact-checking services and official health organization channels on social media [8]. Despite these efforts, combating misinformation remains challenging due to its rapid spread and the persistence of beliefs even after they are debunked [9]. Several studies underscore that misinformation’s impact is not uniform; factors such as cultural attitudes, local media narratives, and socioeconomic conditions influence how individuals interpret and respond to health information [6,10]. These dynamics highlight the importance of examining public health communication within specific geographic contexts. Research focusing on geographic and cultural factors has shown that regional responses to COVID-19 misinformation can vary significantly. For instance, studies in Italy observed differences in how residents in northern versus southern regions perceived and responded to pandemic-related information, with factors such as political ideology, economic disparities, and regional media landscapes contributing to varying levels of trust in official health messages [11]. In the United States, researchers found that COVID-19 misinformation spread differently across urban and rural areas, as well as among different demographic groups, with these variations linked to political affiliations and local media influences [12]. These findings suggest that understanding the spatial and cultural factors in infodemic responses is critical for tailoring effective public health communications. Geographically tagged Twitter data offers a valuable resource for investigating these regional disparities, as it allows researchers to capture real-time public sentiment and discourse based on users’ locations [13]. Analyses of Twitter data have shown how sentiment analysis and topic modeling can identify key themes, concerns, and misinformation trends within specific regions, providing insights into how public health information is received in various cultural and social contexts [14]. However, despite the value of such studies, there remains a gap in understanding how these dynamics play out in regions with diverse socioeconomic and cultural contexts, such as Italy, where regional differences have historically influenced public opinion and media consumption [15,16].

This study builds upon existing research by examining infodemic responses across Italy’s distinct regions, focusing on 10 cities from the north, center, south, and islands. By leveraging Twitter data to analyze public perception and discourse, we aim to reveal how regional factors influence responses to COVID-19 information. Our research contributes to the broader literature by highlighting the importance of geographic context in the study of misinformation, providing insights that can inform more localized and effective public health strategies to address misinformation in future health crises.

## 3. Materials and Methods

### 3.1. Data Collection and Dataset Description

**Figure 1:**
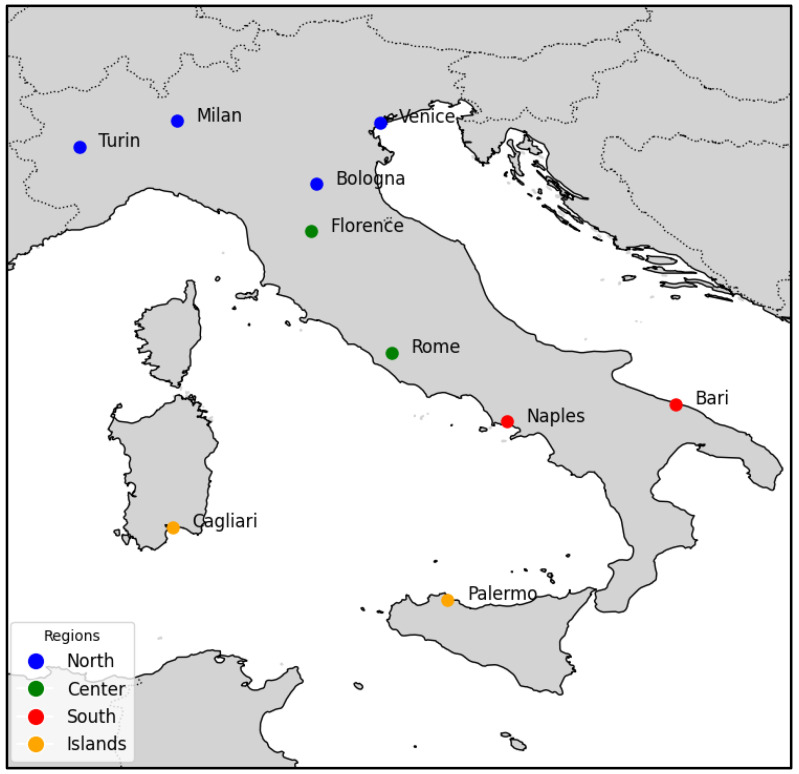
Ten cities map plot (Italy).

**Figure A1:**
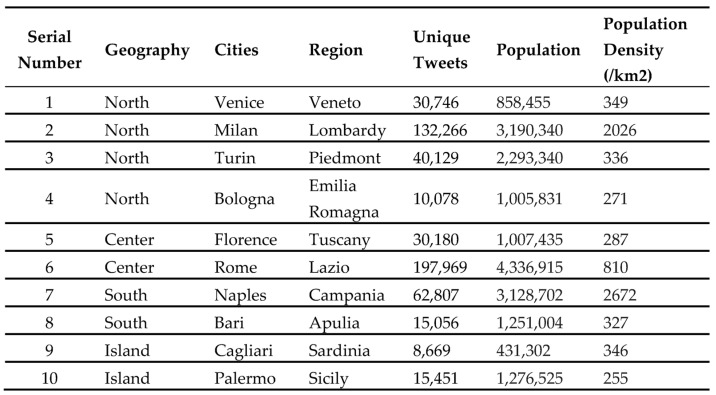
Tweet distribution by city.

The study utilizes a comprehensive dataset comprising 535,886 tweets collected over a ten-month period from 30 August 2020 to 8 June 2021. The data collection strategy was focused on capturing geographical variations in COVID-19 discourse across Italy by focusing on ten strategically selected cities. The following cities were chosen to represent the diverse geographical regions of Italy, including the North: Milan, Turin, Venice, and Bologna; Center: Florence and Rome; South: Naples and Bari; and Islands: Cagliari and Palermo, enabling a nuanced analysis of regional variations in public discourse and information dissemination patterns during the pandemic (See Figure 1). (Refer to Figure A1 in Appendix A to view the distribution and count of tweets analyzed for each city).

The selection of these specific cities was driven by several factors. First, they represent major population centers across different Italian regions, ensuring a substantial volume of social media activity. Second, these cities experienced varying intensities and timelines of COVID-19 impact, providing an opportunity to examine how local context influenced public discourse. Third, the cultural and socioeconomic diversity across these cities enables examination of how regional characteristics might influence information processing and sharing behaviors during the health crisis. The dataset structure was designed to capture multiple dimensions of each tweet, including unique identifiers, user information (screen names, full names, and locations), interaction metadata (retweet status and user mentions), and temporal information (timestamps). This rich data structure allows for both broad pattern analysis and detailed examination of specific interaction dynamics. The temporal span of the dataset is particularly significant as it covers several critical phases of the pandemic in Italy, including various waves of infection, policy changes, safety health measures, and the initial phases of vaccine rollout. Twitter keywords consisted of a set of predefined COVID-19 search key terms in both the English and Italian languages. Predefined Twitter search key terms/hashtags in English included: COVID-19, Coronavirus, CoronavirusOutbreak, coronavirusitaly, racism, COVID2019, COVID19italy, Flu, ItalyCoronavirus, Lombardy, Italyquarantine, quarantineItaly, and COVID. Predefined Twitter search key terms in Italian included: razzismo, Italiani all’estero, Influenza, Amuchina, Codogno, Contagiati, Contagio, Coronaviriusitalia, COVID19italia, COVID2019italia, Coronavirusitalia, CoronavirusItalla, Lombardia, zonarossa, focolai, and quarentena. Tweepy API was used to collect tweets in compliance with Twitter’s terms of service and privacy policies. The study only collected publicly available tweets, excluding private or protected accounts. The dataset did not include sensitive personal information, such as direct messages or user contact details.

### 3.2. Data Preprocessing and Translation

#### Text Translation Strategy

The tweets collected from Twitter were predominantly Italian which necessitated a careful approach to translation. After evaluating various translation options, including specialized machine translation models and professional translation services, Google Translate was selected as the primary translation tool from Italian to English language. This choice was influenced by several practical considerations: the need to process a large volume of tweets cost-effectively, the requirement for consistent translation quality across diverse dialectal variations, and the ability to handle informal language and social media-specific content. To ensure translation quality, a systematic verification process was implemented. This involved manual review of a significant sample of translated tweets by bilingual speakers. The verification process focused particularly on preserving contextual meanings, accurately translating regional expressions, and maintaining the emotional tone of the original messages. This quality control step was crucial for ensuring the reliability of subsequent sentiment and topic analyses.

### 3.3. Network Analysis

#### 3.3.1. Network Construction and Initial Analysis

**Figure 2:**
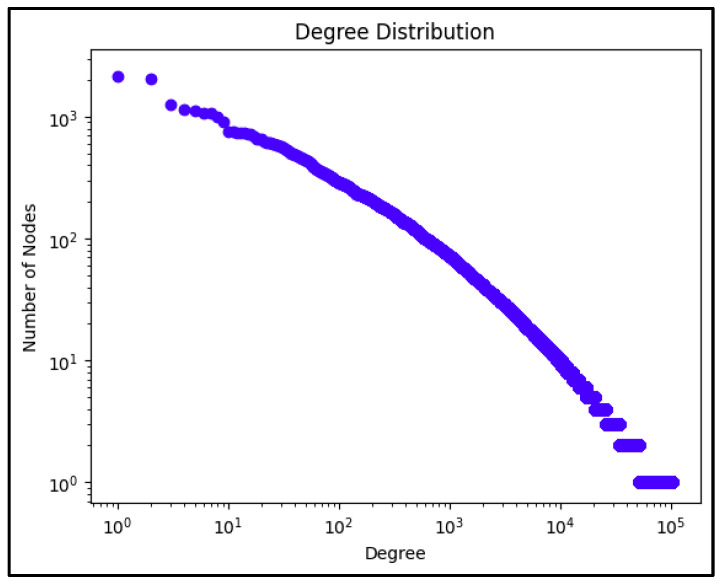
Degree distribution for the entire network.

The construction of the social network representation began with the creation of a directed graph structure where nodes represented individual users, identified by their Twitter screen names, and edges represented meaningful interactions between users. Two types of interactions were considered for edge creation: direct mentions of users within tweets and retweet relationships. This approach resulted in an initial network comprising 103,838 nodes and 287,577 edges, providing a comprehensive representation of the information flow and social interactions within the COVID-19 discourse. The decision to create a directed network rather than an undirected one was driven by the need to preserve the directionality of information flow, which is particularly crucial in understanding how COVID-19-related information and misinformation spread through social networks. A mention or retweet represents a deliberate act of engagement with another user’s content, and the direction of this engagement carries significant meaning in the context of information dissemination (See Figure 2).

Figure 2 shows the initial analysis of the network, which revealed characteristics typical of social media networks, most notably a power–law degree distribution. This distribution pattern indicated that while most users had relatively few connections, a small number of users acted as highly connected hubs within the network. This finding aligns with established research on social media networks and suggests the presence of influential users who played crucial roles in information dissemination during the pandemic.

#### 3.3.2. Network Metrics and Structural Analysis

The structural analysis of the network began with the calculation of several key metrics that provided insight into the network’s organizational characteristics. The clustering coefficient, measured at 0.056, revealed that only about 5.6% of potential connections among a node’s neighbors were actually present. This relatively low clustering coefficient suggested that the network exhibited limited local clustering, indicating that users’ immediate networks were not densely interconnected. The transitivity measure of 0.014 provided further insight into the global clustering patterns, indicating that only 1.4% of potential transitive relationships were realized within the network. This low transitivity score suggested that the network was characterized by loosely connected communities rather than tightly knit clusters, pointing to a more distributed and less hierarchical structure. Such a structure has implications for information flow, as it suggests that information typically traveled through specific pathways rather than being redundantly shared through multiple channels. The network density calculation yielded a value of 2.667139319574774 × 10^−5^, indicating an extremely sparse and distributed network structure. This sparsity is not unusual for large social networks and suggests that users typically maintained connections with a small fraction of the total network population. The low density also implies that information flow within the network was likely controlled by key nodes that bridged otherwise disconnected parts of the network.

#### 3.3.3. Network Filtering and Refinement

The initial analysis revealed the need for network filtering to focus on the most significant interactions and remove noise from the dataset. A systematic approach to filtering was developed by testing various degree thresholds to find an optimal balance between network size and information retention. After extensive testing with different threshold combinations, a minimum degree of 20 and maximum degree of 2451 was selected as the optimal filtering criteria. This filtering process reduced the network to 4792 nodes and 77,785 edges, representing approximately 4.6% of the original nodes but retaining 27.3% of the original edges. The selection of these specific thresholds was based on several considerations. The minimum degree of 20 ensured that only users with substantial engagement in the COVID-19 discourse were included in the analysis. The filtered network maintained the essential structural characteristics of the original network while providing a more focused view of the significant interactions across geographies. This refinement was crucial for the subsequent community detection and influence analysis phases, as it allowed for more meaningful analysis of interaction patterns among consistently engaged users.

#### 3.3.4. Community Detection and Analysis

**Figure 3:**
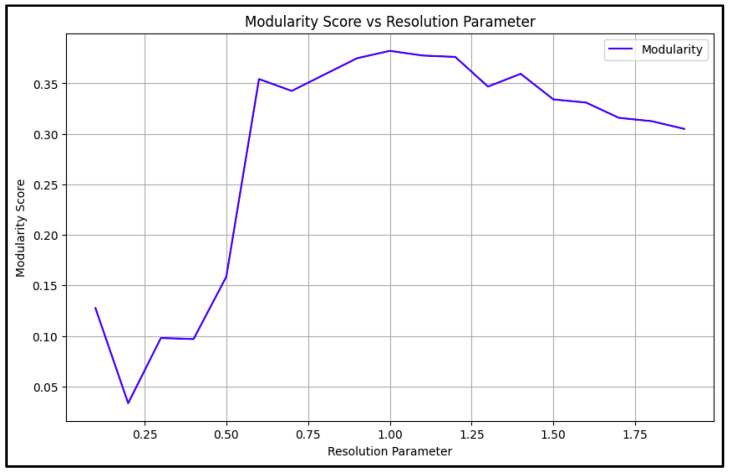
Modularity score vs. resolution parameter, used to find the highest modularity score.

The community detection phase employed the Louvain algorithm, which was chosen for its ability to handle large networks efficiently while producing high-quality community assignments. The algorithm’s resolution parameter was carefully optimized through an extensive search process, testing values from 0.1 to 2.0 to find the optimal balance between community size and modularity (See Figure 3).

**Figure 4:**
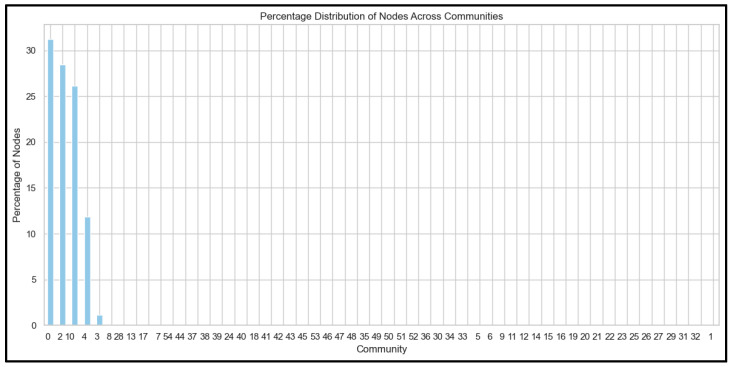
Percentage distribution of nodes across communities (Note: Communities 8 and 28 do not appear due to low node size).

As Figure 3 shows, the optimization process revealed that a resolution parameter of 1.0 produced the most meaningful community structure, resulting in 55 distinct communities across the dataset. This parameter choice was validated through multiple quality metrics. The modularity score of 0.382 indicated significant community structure, surpassing the typical threshold of 0.3 for meaningful community detection. The conductance measure of 0.172 suggested well-defined communities with limited external connections, while the coverage value of 0.694 indicated that a substantial portion of the network’s edges were contained within communities (See Figure 4).

**Figure 5:**
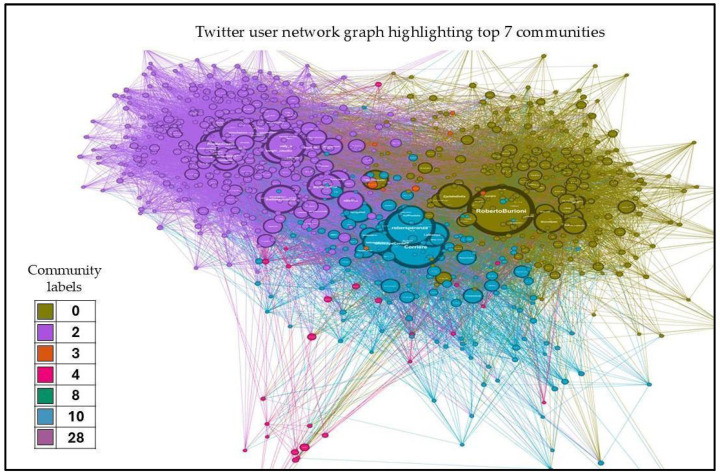
Network graph highlighting the top seven communities (high degree nodes).

Further analysis shown in Figure 4 revealed that the top seven communities contained 98.9% of the nodes, with detailed percentage distributions as follows: Community 0 contained 31.239566% of nodes, Community 2 held 28.484975%, Community 10 comprised 26.126878%, Community 4 contained 11.832220%, Community 3 held 1.168614%, and Communities 8 and 28 each contained 0.041736% of nodes. This highly skewed distribution suggested a concentration of key influential users in the top communities with the highest percentage distribution of nodes in the network. Figure 5 shows the network graph with communities with high degree nodes.

#### 3.3.5. Influence Analysis and Key User Identification

The identification of influential users within the network was approached through two complementary centrality measures: degree centrality and betweenness centrality. Degree centrality was used to identify users with the highest number of direct connections, representing locally influential nodes within the network. These users typically acted as primary sources or amplifiers of information within their immediate network neighborhood. Betweenness centrality analysis identified users who served as bridges between different parts of the network. These users were particularly important as they often controlled the flow of information between otherwise disconnected communities. The combination of these two centrality measures provided a comprehensive view of influence within the network, capturing both local and global aspects of information flow.

### 3.4. Misinformation Analysis

#### 3.4.1. Development of Misinformation Detection Framework

The analysis of potential COVID-19 misinformation required the development of a systematic framework for identifying and analyzing suspicious content within the dataset. This framework began with the careful curation of a specialized keyword lexicon comprising 48 terms and phrases frequently associated with COVID-19 misinformation, obtained from the literature [7,17,18,19]. The selection of these keywords, as shown in Table 1, was informed by multiple sources, including the academic literature on COVID-19 misinformation, fact-checking organizations’ reports, and documented patterns of pandemic-related conspiracy theories. The selection process paid particular attention to terms that had demonstrated strong associations with vaccine hesitancy, alternative treatment promotion, policy skepticism, and conspiracy theories related to the origin and spread of the virus. The keywords were carefully chosen to balance sensitivity (ability to catch potential misinformation) with specificity (avoiding false positives from legitimate scientific discourse).

#### 3.4.2. Community-Level Misinformation Analysis

The application of the misinformation detection framework at the community level revealed distinct patterns in how different network communities engaged with and shared potentially misleading information. This analysis was particularly important given the identification of seven major communities that collectively contained 98.9% of the network nodes. The distribution of suspicious keywords across these communities provided insight into how different sub-groups within the Italian social media landscape approached and processed pandemic-related information. The analysis examined not only the frequency of suspicious keyword appearances, but also the context in which they appeared. This contextual analysis was crucial for distinguishing between legitimate discussion of misinformation (such as fact-checking or news reporting) and actual promotion of misleading narratives.

#### 3.4.3. User-Level Misinformation Analysis

The analysis of misinformation patterns among the top 100 influential users, identified through centrality measures, provided crucial insights into the role of key network actors in either promoting or countering misinformation. This focused analysis examined how users with a high degree of centrality and betweenness centrality engaged with potentially misleading content, considering both their original posts and their interactions with other users’ content. The investigation considered several factors, including the frequency of suspicious keyword usage, the context of such usage, the temporal patterns of engagement with potentially misleading content, and the relationship between a user’s influence metrics and their propensity to engage with suspicious content. This multi-dimensional analysis helped understand how influential users might have shaped the information environment during the pandemic.

### 3.5. Tools and Software Implementation

The research methodology was implemented through a carefully selected suite of software tools, with Python 3.11 serving as the primary programming environment. The data management aspects of the research were handled through the Pandas library version 2.1.1 [20]. The NumPy library version 1.26.0 [21] supported the numerical computations. The NetworkX library version 3.2.1 [22] provided the core functionality for network analysis, offering a comprehensive set of tools for creating, manipulating, and analyzing complex network structures. Gephi 0.10.1 [23] was used to develop network graph visualizations and perform exploratory analysis. Visualization of results was accomplished through a combination of Matplotlib version 3.9.0 [24] and Seaborn version 0.13.2 [25] libraries.

## 4. Results

### 4.1. Community Exploration and Analysis of 10 Italian Cities

**Figure 6:**
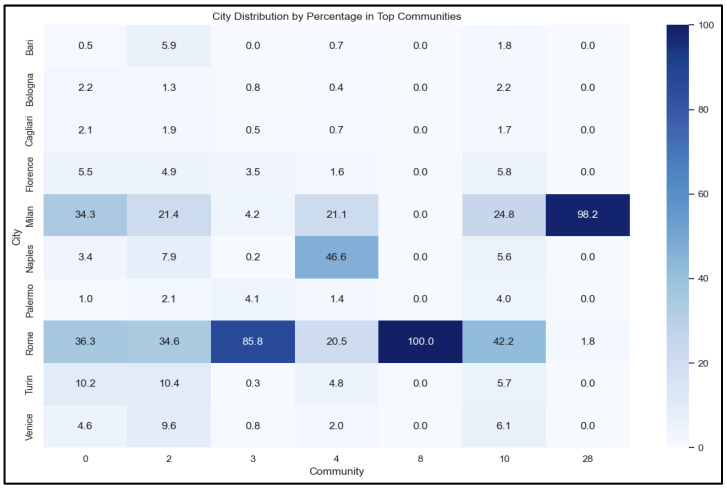
Distribution of nodes across the top seven communities identified during the network analysis for 10 Italian cities by percentage in the top seven communities.

Figure 6 below visualizes the distribution of nodes across the top communities identified during the network analysis for 10 Italian cities. Each city’s involvement in various communities is expressed as a percentage, highlighting the extent of their connections and influence within these groups.

This analysis follows the major events during the COVID-19 pandemic and helps uncover patterns in misinformation dissemination and engagement, revealing the cities most affected by community-specific narratives which is presented in Table 2. Contextual analysis was performed by reviewing a sample of tweets from each community. This provided nuanced insights into how specific keywords were used and also revealed potential thematic connections and predominant narratives. (Please refer to the website in the Supplementary Information section to view the major events/policy timeline gathered from news headlines during the COVID-19 pandemic and a sample of tweets from each community).

### 4.2. Community-Level Misinformation Analysis

**Figure 7:**
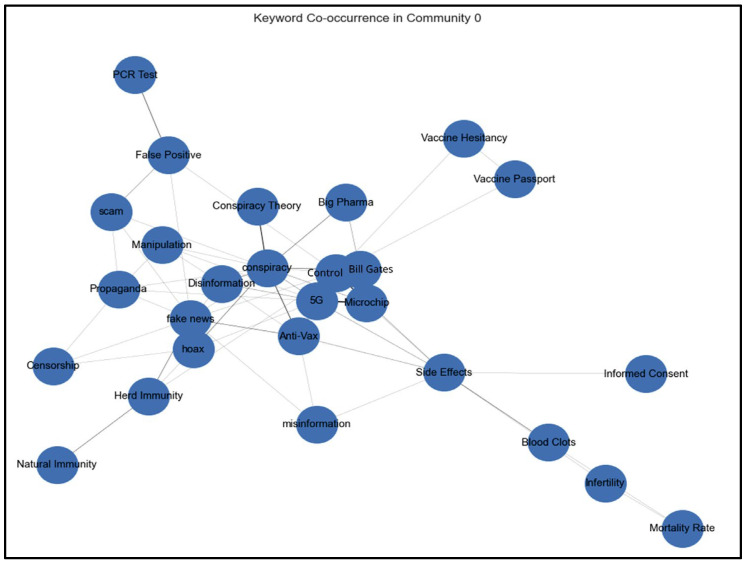
Community 0 keyword co-occurrence.

To analyze misinformation, tweets containing suspicious keywords were grouped by community for detailed examination. Co-occurrence patterns of keywords within tweets were identified to uncover frequently paired terms, shedding light on interconnected narratives. Keyword co-occurrence networks, visualized below for specific communities, highlight keyword relationships and potential echo chambers. These keyword co-occurrence networks are used to understand patterns of keyword use and the dynamics of information propagation across communities. Figure 7 shows the community-level misinformation analysis of Community 0 keyword co-occurrence.

Analysis of the keyword co-occurrence network in Community 0 reveals distinct echo chambers where COVID-19 misinformation narratives were reinforced through repeated patterns of discussion. As shown in Figure 7, at the network’s core, a dense cluster connects “conspiracy”, “5G”, “Bill Gates”, and “microchip”, indicating the prevalence of technological control narratives. A secondary chain linking “Blood Clots”, “Infertility”, and “Mortality Rate” reflects the amplification of vaccine safety concerns. Information control narratives emerge through the interconnection of “Censorship”, “Manipulation”, “Propaganda”, and “Disinformation”, all tied to “fake news” and “hoax” keywords. The network also shows a branch of medical authority skepticism, where “PCR Test” connects to “False Positive” and “scam”. These patterns, observed in content from Milan, Rome, and Turin, demonstrate how users within Community 0 were exposed to and perpetuated specific conspiracy narratives through tightly clustered, self-reinforcing information channels with limited exposure to alternative viewpoints or fact-based discussions.

**Figure 8:**
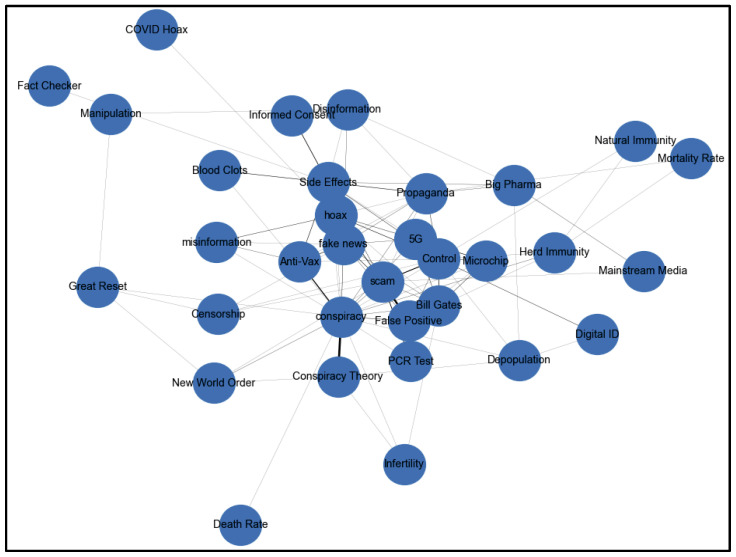
Community 2 keyword co-occurrence.

Figure 8 shows the community-level misinformation analysis of Community 2 keyword co-occurrence.

The keyword co-occurrence network (Figure 8) for Community 2 reveals a complex web of interconnected misinformation narratives centered around COVID-19. At its core, the network shows three distinct but interrelated echo chambers: a pandemic control narrative linking “Bill Gates”, “5G”, and “microchip”; a medical misinformation cluster connecting “Side Effects”, “Blood Clots”, and “Anti-Vax”; and an institutional distrust group involving “Big Pharma”, “Mainstream Media”, and “Fact Checker”. These clusters are bound together by broader conspiracy frameworks like “New World Order” and “Great Reset”, suggesting that community members engaging with one piece of misinformation are likely to encounter related misleading narratives. The dense interconnections between these themes indicate a self-reinforcing information environment where alternative viewpoints may struggle to penetrate, creating a classic echo chamber effect where existing beliefs are continuously reinforced through repeated exposure to similar narratives.

**Figure 9:**
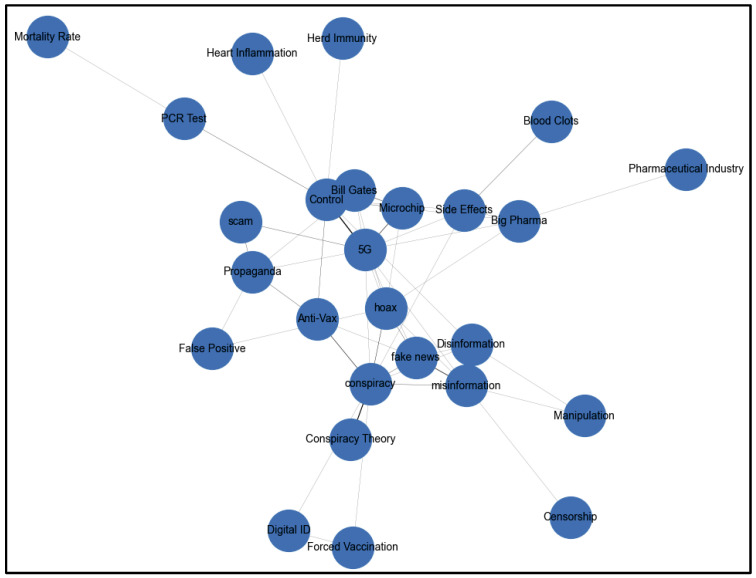
Community 10 keyword co-occurrence.

Figure 9 shows the community-level misinformation analysis of Community 10 keyword co-occurrence.

The keyword co-occurrence network (Figure 9) in Community 10 displays a more focused and less densely interconnected pattern of misinformation compared to Community 2. The network centers primarily around two key nodes—“5G” and “Bill Gates Control”—which connect to various health-related concerns and institutional distrust narratives. The conspiracy elements in this community appear more technology-oriented, with strong connections between “5G”, “Microchip”, and control-related narratives. Unlike Community 2, this network shows fewer broad conspiracy frameworks and instead concentrates on specific technological and medical concerns. The connection patterns between “Disinformation”, “fake news”, and “conspiracy” appear more peripheral, indicating that this community may be more focused on specific concerns about technological control and medical risks rather than embracing a wider range of conspiracy theories. This structure suggests that the discussions in this community are focused on general discussions at the technology–health intersection, particularly around vaccination and digital control narratives, rather than a specific narrative of spreading misinformation.

**Figure 10:**
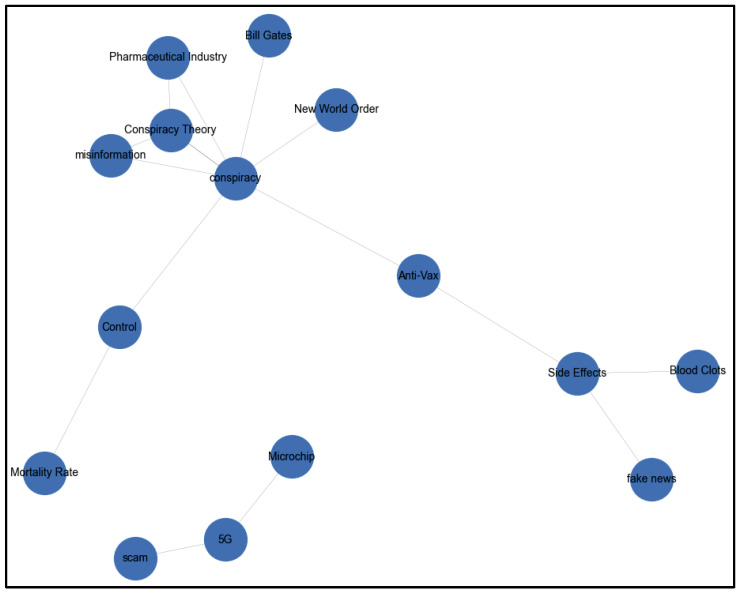
Community 4 keyword co-occurrence.

Figure 10 shows the community-level misinformation analysis of Community 4 keyword co-occurrence.

Community 4’s keyword co-occurrence network (Figure 10) reveals a notably sparse and linear structure, contrasting sharply with the dense interconnections seen in Communities 2 and 10. The network appears to split into two main branches, with “conspiracy” serving as the central node. One branch connects to institutional themes, linking “Conspiracy Theory”, “Pharmaceutical Industry”, “Bill Gates”, and “New World Order”, suggesting a narrative focused on institutional control. The other branch extends through “Anti-Vax” to medical concerns like “Side Effects” and “Blood Clots”, with a separate sub-branch connecting “5G”, “Microchip”, and “scam”. This linear, less interconnected structure suggests that Community 4 may represent a less established echo chamber, where narratives flow in a more sequential rather than reinforcing pattern. The reduced density of connections could indicate that this community’s members engage with specific strands of misinformation rather than embracing a fully integrated conspiracy worldview, making it potentially more amenable to factual information that addresses individual concerns.

### 4.3. Influential User Misinformation Assessment

#### 4.3.1. Influential User Misinformation Trend Analysis

**Figure 11:**
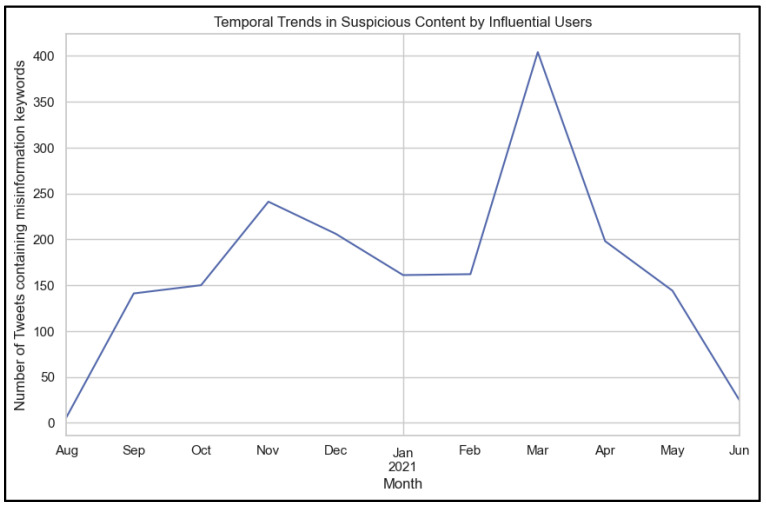
Temporal trends by influential users (Blue line represents the number of tweets containing misinformation keywords).

Figure 11 below illustrates the temporal trends of tweets containing suspicious or misinformation-related keywords posted by influential users. The number of tweets containing misinformation related keywords, illustrated as a blue line in Figure 11 below, first peaks in November. This period likely coincides with heightened public discourse around the second wave of COVID-19 in Italy, including debates about restrictions, vaccinations, and pandemic policies. Activity levels dropped slightly in December and remained relatively stable through February. This decline could be attributed to the holiday season or a reduction in pandemic-related announcements and misinformation circulation during this period. A dramatic surge occurred in March 2021, possibly linked to significant events, such as the rollout of vaccines or stricter lockdown measures in Italy. This period might have triggered polarizing opinions and misinformation among influential users. Peaks in activity, fear-mongering, and the spread of misinformation align with periods of heightened uncertainty, significant policy changes, or public anxiety, reflecting how influential users amplified contentious narratives during critical phases of the pandemic.

#### 4.3.2. Overview of Selected Influential User by Community

Analysis of tweet patterns among users in Communities 0, 2, and 10 revealed several key influential figures who shaped the discourse within their respective networks (refer to the website in the Supplementary Information to view a sample of tweets from influential users).

Community 0 shows an interesting dynamic between pro-vaccine and anti-vaccine voices. Notable pro-vaccine influential users include ‘Roberto Burioni’, a virologist known for public health advocacy, who consistently shared evidence-based information about vaccine safety and effectiveness, particularly focusing on addressing concerns about side effects and emphasizing the importance of youth vaccination. ‘Ricardo Puglisi’, an economist, and ‘Daniele Dellavedova’, a politician, both supported vaccination efforts while maintaining critical perspectives on implementation. However, the community also featured prominent anti-vaccine voices like ‘stanzaselvaggia’, who frequently shared posts questioning lockdown measures and vaccine efficacy, often using provocative language and sarcasm to undermine public health messaging.

Community 2 presents a more polarized landscape. On the pro-vaccine side, ‘Myrta Merlino’, a TV anchor, and ‘Ettore Giuliano’, a biologist, focused on promoting public health measures and vaccine acceptance. However, this community showed stronger anti-vaccine sentiment through users like ‘embonaccorso’ and ‘BarillariDav’. ‘BarillariDav’, notably a politician in the governing party, frequently questioned the very existence of the pandemic, using hashtags like #covid1984 and promoting conspiracy theories. Embonaccorso’s tweets actively discouraged vaccination, particularly for children, and spread misinformation about vaccine safety and effectiveness.

Community 10 presents an interesting contrast between traditional news reporting and misinformation narratives. The network analysis reveals that while conspiracy-related keywords were present, this community was notably influenced by established news organizations providing factual coverage of the pandemic. Major news outlets like LaStampa, Corriere, and Agenzia Italia played a significant role in shaping the community’s information environment. The frequency of them reporting on tweets focused on concrete events and developments.

## 5. Discussion

The findings of this study underscore the complex dynamics of misinformation during the COVID-19 pandemic, particularly in the Italian context. By leveraging geographically tagged Twitter data, our analysis highlights the intricate interplay between regional characteristics, public discourse, and misinformation narratives. The results reveal across the seven communities to show significant variations in the themes, intensity, and patterns of misinformation across different Italian communities, reflecting broader sociocultural, economic, and political factors.

Our study identifies notable differences in the spread and impact of misinformation across northern, central, southern and island regions of Italy. Communities in northern Italy, such as Milan and Turin, were significantly impacted by economic hardships and healthcare disparities, fueling conspiracy theories about government overreach and vaccine safety. These findings align with previous studies such as those by [4,6,7], which observed how economic and healthcare inequalities can exacerbate misinformation uptake. On the other hand, communities in southern Italy, such as Naples and Palermo, exhibited unique narratives centered around unverified treatments and alternative remedies. These regional differences reflect longstanding socioeconomic disparities and cultural variations, which may influence trust in institutions and are prone to misinformation. Similar patterns have been observed in prior research studies, which highlighted regional divides in public health messaging efficacy and trust in governmental policy directives. To understand the echo chambers and network dynamics, the study conducted a keyword co-occurrence networks analysis which revealed distinct echo chambers within seven communities, characterized by tightly clustered misinformation narratives that were spread during the pandemic. Communities 0 and 2 exhibited dense interconnections between conspiracy theories, vaccine safety concerns, and institutional distrust. In major urban centers with stricter vaccine policies, misinformation beacons like “herd immunity”, “natural immunity”, and “microchip” were prominent, reflecting pseudo-scientific narratives. Keywords such as “Bill Gates”, “5G”, and “forced vaccination” highlighted widespread conspiracies, particularly in anti-vaccine circles. This self-reinforcing structure highlights misinformation, creating environments resistant to corrective information. Community 4 displayed a more linear and less interconnected structure, suggesting a nascent echo chamber. These findings are significant as they indicate that less dense misinformation networks may be more receptive to targeted public health interventions. Prior research has emphasized the role of echo chambers in applying misinformation, and our results contribute to this understanding by providing a granular analysis of community-level variations across Italy.

## 6. Conclusions

The thematic diversity observed across communities ranging from vaccine safety and quarantine regulations to conspiracy theories and economic concerns highlights the need for tailored public health communities strategies. Our findings suggest that geographic and cultural factors significantly shape public discourse and risk perception, necessitating localized approaches to misinformation mitigation. For instance, in Milan and Rome, where economic and political narratives dominate, messaging could focus on addressing specific concerns about economic recovery and government transparency while tailoring and monitoring vaccination education campaigns. In southern areas of Italy such as Naples, efforts should emphasize building trust through community-led initiatives and addressing cultural beliefs about health practices. Analysis of top influencers in the network revealed a clear dichotomy in how information was shared during the pandemic. Established public figures—including medical professionals, journalists, and academics—consistently provided evidence-based information and practical guidance, helping to anchor public understanding during uncertain times. In contrast, a distinct group of influential users emerged who actively spread misinformation about vaccines and government policies. These users skillfully cultivated followings by promoting conspiracy theories and anti-establishment narratives, successfully creating small echo chambers where their skeptical views could flourish and gain prominence. This split among influential voices highlighted the competing narratives that shaped public discourse throughout the pandemic crisis.

By illuminating the regional nuances of misinformation during the COVID-19 pandemic, this study provides valuable insights into the mechanisms of misinformation propagation and its impact on public health communication. The findings emphasize the need for targeted, context specific strategies to combat misinformation and enhance community resilience during health crises. The insights gained from such studies can significantly aid in shaping future pandemic prevention and intervention policies. Public health agencies must consider geographic, cultural, and socioeconomic factors when designing interventions to ensure effective communication and misinformation mitigation. Future research should explore the longitudinal effect of misinformation on public behavior and trust in institutions, particularly in the context of vaccine uptake and compliance with health measures. Finally, expanding this analysis to other regions and platforms could provide a more comprehensive understanding of the global infodemic which could uncover universal patterns and unique challenges in combating misinformation.

## 7. Limitations and Future Work

While this study provides valuable insights into regional variations in COVID-19 discourse and misinformation patterns, it is not without limitations. These limitations highlight opportunities for future research to build upon and expand the findings presented here.

### 7.1. Reliance on Geotagged Tweets

The study relies exclusively on geotagged tweets, which represent a small fraction of all tweets and may introduce selection bias. Users who enable geotagging may differ from the broader Twitter population in terms of demographics, behavior, or motivations for sharing location data. Future research could explore alternative methods for inferring location, such as analyzing user profile information or leveraging machine learning models to predict geographic locations based on tweet content.

### 7.2. Regional and Temporal Scope

The study focuses on 10 Italian cities over a ten-month period, which may limit the generalizability of the findings to other regions or timeframes. While the analysis captures key phases of the pandemic in Italy, future research could expand the geographic and temporal scope of the analysis to include additional regions and time periods, providing a more comprehensive understanding of how misinformation evolves over time and across contexts.

### 7.3. Influence of Algorithmic Bias

Social media platforms like Twitter are influenced by algorithmic bias, which affects the visibility and dissemination of content [26]. The study does not account for how Twitter’s algorithms may prioritize certain types of content over others, potentially skewing the dataset. Future research could explore the role of algorithmic bias in shaping misinformation patterns and develop methods to control for these effects in the analysis. Moreover, these studies can also support the implementation of pandemic management strategies and policies from the point of view of compliance and feedback.

## Figures and Tables

**Table 1:** 48 COVID-19 misinformation keywords for lexical search [7,17,18,19].

48 COVID-19 Misinformation Keywords for Lexical Search						
5G	Alternative Media	Anti-Vax	Big Pharma	Bill Gates	Blood Clots	Bodily Autonomy
Casedemic	Censorship	Conspiracy	Conspiracy Theory	Control	COVID Hoax	Death Rate
Depopulation	Digital ID	Disinformation	DNA Damage	Fact Checker	Fake News	False Positive
Forced Vaccination	Globalist Agenda	Government Conspiracy	Great Reset	Health Freedom	Heart Inflammation	Herd Immunity
Hoax	Hospitalization Rate	Infertility	Informed Consent	Mainstream Media	Manipulation	Medical Tyranny
Microchip	Misinformation	Mortality Rate	Natural Immunity	New World Order	PCR Test	Pharmaceutical Industry
Propaganda	Scam	Side Effects	Surveillance State	Vaccine Hesitancy	Vaccine Passport	

**Table 2:** Observed spread of misinformation across communities through tweet discourse.

Community	Major Cities	Spread of Misinformation
Community 0	Milan, Rome, Turin	In Milan, economic hardship and religious restrictions fueled conspiracy theories, while worker protests amplified anti-government rhetoric. Rome faced misinformation around vaccination policies and cultural restrictions, sparking public distrust. In Turin, healthcare inequities and vaccine safety concerns were exacerbated by digital misinformation during lockdowns.
Community 2	Milan, Rome, Venice, Turin	Northern Italy, particularly Milan and Turin, bore the brunt of COVID-19’s early impact. The cancellation of religious events, police enforcement of restrictions, and economic turmoil fueled conspiracy theories. Venice, which is reliant on tourism, saw misinformation about government policies and virus severity, deepening economic anxieties.
Community 10	Milan, Rome	Milan and Rome, as major centers of economic, political, and religious significance, emerged as key hotspots for misinformation. While prominent news outlets consistently shared accurate updates on vaccine development, COVID-19 mortality rates, and other critical topics, false narratives about vaccine side effects and claims of government propaganda gained traction among certain user groups, exacerbating public skepticism. Despite their advanced healthcare resources, these cities faced challenges with compliance, driven largely by the pervasive atmosphere of distrust.
Community 4	Naples, Rome, Milan	Naples, Rome, and Milan navigated misinformation differently due to their distinct cultural contexts. Naples saw fear and confusion around unverified treatments, while Milan contended with economic unrest and Rome faced anti-vaccine campaigns tied to political narratives. Shared acts of solidarity countered misinformation to some extent.
Community 3	Rome, Palermo, Florence	Rome, Palermo, and Florence encountered misinformation about virus transmission, vaccine safety, and alternative treatments. False narratives like #IoNonMiVaccino and #DittaturaSanitaria led to public confusion, non-compliance, and vulnerability among marginalized groups.
Community 8	Rome	Rome saw the spread of conspiracies about 5G and government overreach, disproportionately affecting the elderly and economically vulnerable. Misleading hashtags amplified fears, leading to non-compliance with health measures and setbacks in tourism and small businesses.
Community 28	Milan	Milan’s journey through the pandemic included misinformation-driven panic during early lockdowns and resistance to vaccination campaigns. However, the city counteracted these with resilience through public health campaigns, creative social initiatives, and a gradual return to normality by 2022.

**Figure A2:**
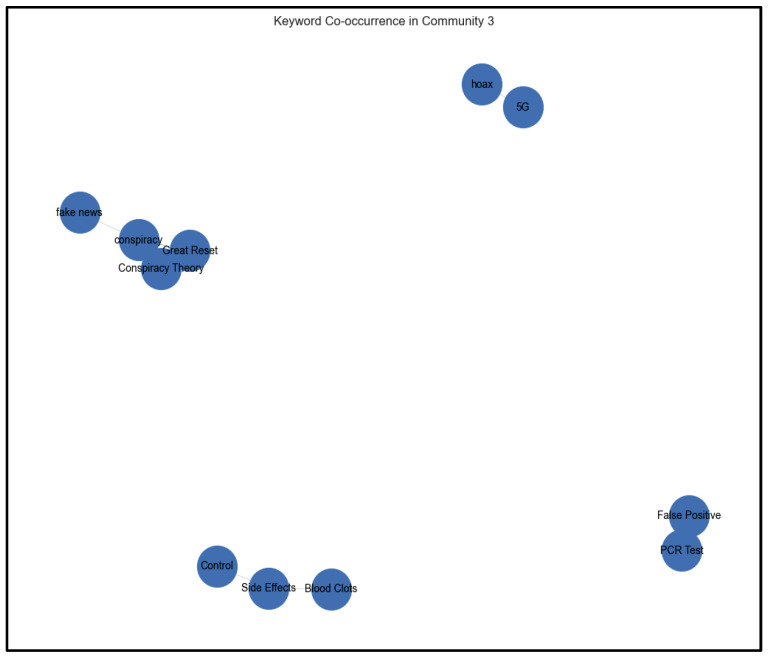
Community 3 keyword co-occurrence.

**Figure A3:**
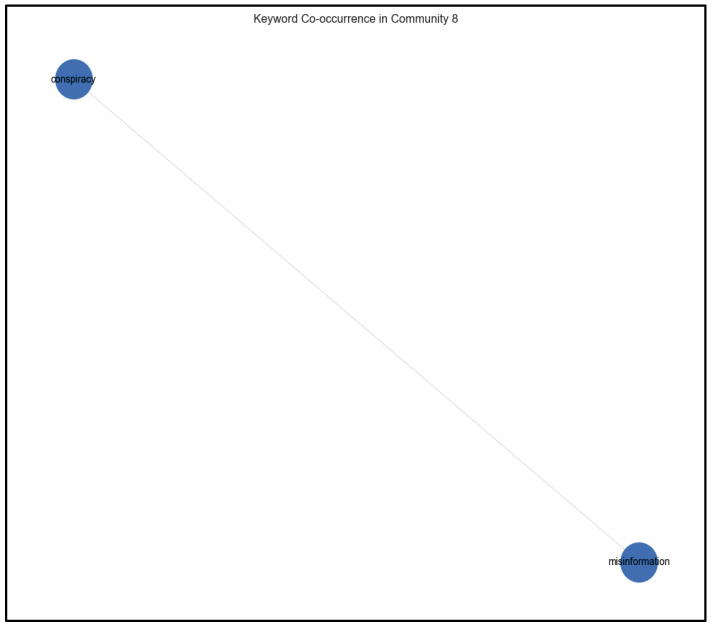
Community 8 keyword co-occurrence.

## Data Availability

Data are available upon request; refer to the website for the contact information.

## Supplementary Materials

The following supporting information and the data can be downloaded at the webpage: https://sites.google.com/view/networkanalysis-covid19italy/home?authuser=0 (accessed on 9 April 2025).

## Appendix A

### Community-Level Misinformation

Community 3: Rome, Palermo, and Florence encountered misinformation about virus transmission, vaccine safety, and alternative treatments. False narratives like #IoNonMiVaccino and #DittaturaSanitaria led to public confusion, non-compliance, and vulnerability among marginalized groups.

## References

[1] Cinelli M., Quattrociocchi W., Galeazzi A., Valensise C.M., Brugnoli E., Schmidt A.L., Zola P., Zollo F., Scala A. (2020). The COVID-19 social media infodemic. Sci. Rep..

[2] Pulido C.M., Villarejo-Carballido B., Redondo-Sama G., Gómez A. (2020). COVID-19 infodemic: More retweets for science-based information on coronavirus than for false information. Int. Sociol..

[3] Gallotti R., Valle F., Castaldo N., Sacco P., De Domenico M. (2020). Assessing the risks of ‘infodemics’ in response to COVID-19 epidemics. Nat. Hum. Behav..

[4] Rovetta A., Castaldo L. (2021). Influence of Mass Media on Italian Web Users During the COVID-19 Pandemic: Infodemiological Analysis. JMIRx Med..

[5] Eysenbach G. (2002). Infodemiology: The epidemiology of (mis)information. Am. J. Med..

[6] De Clerck B., Fernandez Toledano J.C., Van Utterbeeck F., Rocha L.E.C. (2024). Detecting coordinated and bot-like behavior in Twitter: The Jürgen Conings case. EPJ Data Sci..

[7] Roozenbeek J., Schneider C.R., Dryhurst S., Kerr J., Freeman A.L.J., Recchia G., van der Bles A.M., van der Linden S. (2020). Susceptibility to misinformation about COVID-19 around the world. R. Soc. Open Sci..

[8] Chen L., Chen J., Xia C. (2021). Social network behavior and public opinion manipulation. J. Inf. Secur. Appl..

[9] Brennen J.S., Simon F., Howard P.N., Nielsen R.K. (2020). Types, Sources, and Claims of COVID-19 Misinformation. Reuters Institute for the Study of Journalism. https://reutersinstitute.politics.ox.ac.uk/types-sources-and-claims-covid-19-misinformation.

[10] Allington D., Dhavan N. (2020). The Relationship Between Conspiracy Beliefs and Compliance with Public Health Guidance with Regard to COVID-19.

[11] Fernandez G., Maione C., Yang H., Zaballa K., Bonnici N., Carter J., Spitzberg B.H., Jin C., Tsou M.-H. (2022). Social Network Analysis of COVID-19 Sentiments: 10 Metropolitan Cities in Italy. Int. J. Environ. Res. Public Health.

[12] Aiello A.E., Renson A., Zivich P.N. (2020). Social Media–and Internet-Based Disease Surveillance for Public Health. Annu. Rev. Public Health.

[13] Huang X., Li Z., Jiang Y., Li X. (2020). Public perception of the Covid-19 pandemic on Twitter: Sentiment analysis and topic modeling study. JMIR Public Health Surveill..

[14] Fernandez G., Maione C., Zaballa K., Bonnici N., Spitzberg B.H., Carter J., Yang H., McKew J., Bonora F., Ghodke S.S. (2021). The Geography of COVID-19 Spread in Italy Using Social Media and Geospatial Data Analytics. Int. J. Intell. Secur. Public Aff..

[15] Zaballa K., Fernandez G., Maione C., Bonnici N., Carter J., Vito D., Tsou M.-H., Aloulou H., Abdulrazak B., de Marassé-Enouf A., Mokhtari M. (2022). Social Response to COVID-19 SMART Dashboard: Proposal for Case Study. Participative Urban Health and Healthy Aging in the Age of AI.

[16] Fernandez G., Maione C., Yang H., Zaballa K., Bonnici N., Carter J., Tsou M.-H. (2023). COVID-19 Societal Effects and Perceptions: A Case Study of Italy. Med. Sci. Forum.

[17] Singh K., Lima G., Cha M., Cha C., Kulshrestha J., Ahn Y.-Y., Varol O. (2022). Misinformation, believability, and vaccine acceptance over 40 countries: Takeaways from the initial phase of the COVID-19 infodemic. PLoS ONE.

[18] Agley J., Xiao Y. (2021). Misinformation about COVID-19: Evidence for differential latent profiles and a strong association with trust in science. BMC Public Health.

[19] Ismail H., Hussein N., Elabyad R., Abdelhalim S., Elhadef M. (2023). Aspect-based classification of vaccine misinformation: A spatiotemporal analysis using Twitter chatter. BMC Public Health.

[20] McKinney W. Data structures for statistical computing in python. Proceedings of the 9th Python in Science Conference.

[21] Harris C.R., Millman K.J., van der Walt S.J., Gommers R., Virtanen P., Cournapeau D., Wieser E., Taylor J., Berg S., Smith N.J. (2020). Array programming with NumPy. Nature.

[22] Hagberg A.A., Schult D.A., Swart P.J. Exploring network structure, dynamics, and function using NetworkX. Proceedings of the Python in Science Conference.

[23] Bastian M., Heymann S., Jacomy M. (2009). Gephi: An open source software for exploring and manipulating networks. Proc. Int. AAAI Conf. Web Soc. Media.

[24] Hunter J.D. (2007). Matplotlib: A 2D graphics environment. Comput. Sci. Eng..

[25] Waskom M.L. (2021). seaborn: Statistical data visualization. J. Open Source Softw..

[26] Metzler H., Garcia D. (2023). Social Drivers and Algorithmic Mechanisms on Digital Media. Perspect. Psychol. Sci..

